# A Systematic Review of Amenable Resilience Factors That Moderate and/or Mediate the Relationship Between Childhood Adversity and Mental Health in Young People

**DOI:** 10.3389/fpsyt.2018.00230

**Published:** 2018-06-19

**Authors:** Jessica Fritz, Anne M. de Graaff, Helen Caisley, Anne-Laura van Harmelen, Paul O. Wilkinson

**Affiliations:** ^1^Department of Psychiatry, University of Cambridge, Cambridge, United Kingdom; ^2^Department of Clinical, Neuro- and Developmental Psychology, Vrije Universiteit Amsterdam, Amsterdam, Netherlands; ^3^Cambridgeshire and Peterborough NHS Foundation Trust, Cambridge, United Kingdom; ^4^Collaboration for Leadership in Applied Health Research and Care East of England, National Institute for Health Research, Cambridge, United Kingdom

**Keywords:** resilience factors, protective factors, childhood adversity, psychopathology, mental health disorders

## Abstract

**Background:** Up to half of Western children and adolescents experience at least one type of childhood adversity. Individuals with a history of childhood adversity have an increased risk of psychopathology. Resilience enhancing factors reduce the risk of psychopathology following childhood adversity. A comprehensive overview of empirically supported resilience factors is critically important for interventions aimed to increase resilience in young people. Moreover, such an overview may aid the development of novel resilience theories. Therefore, we conducted the first systematic review of social, emotional, cognitive and/or behavioral resilience factors after childhood adversity.

**Methods:** We systematically searched Web of Science, PsycINFO, and Scopus (e.g., including MEDLINE) for English, Dutch, and German literature. We included cohort studies that examined whether a resilience factor was a moderator and/or a mediator for the relationship between childhood adversity and psychopathology in young people (mean age 13–24). Therefore, studies were included if the resilience factor was assessed prior to psychopathology, and childhood adversity was assessed no later than the resilience factor. Study data extraction was based on the STROBE report and study quality was assessed with an adapted version of Downs and Black's scale. The preregistered protocol can be found at: http://www.crd.york.ac.uk/PROSPERO/display_record.asp?ID=CRD42016051978.

**Results:** The search identified 1969 studies, of which 22 were included (eight nationalities, study sample *n* range: 59–6780). We found empirical support for 13 of 25 individual-level (e.g., high self-esteem, low rumination), six of 12 family-level (e.g., high family cohesion, high parental involvement), and one of five community-level resilience factors (i.e., high social support), to benefit mental health in young people exposed to childhood adversity. Single vs. multiple resilience factor models supported the notion that resilience factors should not be studied in isolation, and that interrelations between resilience factors should be taken into account when predicting psychopathology after childhood adversity.

**Conclusions:** Interventions that improve individual, family, and/or social support resilience factors may reduce the risk of psychopathology following childhood adversity. Future research should scrutinize whether resilience factors function as a complex interrelated system that benefits mental health resilience after childhood adversity.

## Introduction

Up to half of Western children and adolescents suffer from at least one type of childhood adversity [CA (1)]. CAs span a wide range of traumatic and stressful experiences, and are associated with an increased risk for subsequent psychopathology (1, 2). Recently, a World Health Organization study, based on data from 21 countries (*N* = 51945), showed that approximately 30% of all mental health problems are attributable to CA (2). Fortunately, not all individuals who have experienced CA develop psychopathology (1, 2). Some remain mentally healthy, succumb shortly but recover quickly, recover in the longer term, or even grow mentally after CA (3–7). These individuals may possess or acquire skills and resources that help them to adapt effectively after CA, a phenomenon known as resilience (3, 5, 8, 9). A better understanding of what sets these individuals apart is critically important for interventions aimed to increase resilience in those with a history of CA.

Resilience is an adaptive process following adversity, and can only be scrutinized when risk has been present (4, 5, 7, 10–14). Moreover, resilience should be considered as a dynamic and changing concept, not as a static trait (3, 5, 7, 8, 11, 13–21). Finally, given that resilient functioning waxes and wanes, it can be improved by resilience enhancing factors [RFs (3, 5, 11, 16, 22, 23)].

RFs have a promotive impact on the adjustment process following CA and thus help individuals to adapt and recover from the sequelae of CA (5, 22, 23). Statistically, RFs operate as a moderator (11, 23), and/or as a positive mediator (13, 24) for the relationship between CA and psychopathology. A moderating RF will operate by lowering the level of psychopathology more in adolescents with CA, compared to adolescents without CA. A mediating RF will mitigate the relationship between CA and psychopathology; if the relationship between CA and the RF has the same directionality as the relationship between the RF and psychopathology, improving the level of the RF would lower the level of psychopathology. To date, some reviews provided overviews of potential RFs (16, 25–27). Yet, these reviews were not specific to adversity in childhood (26), examined one type of CA [e.g., childhood sexual abuse (16, 25, 28)], examined one type of psychopathology [e.g., posttraumatic stress disorder (26, 28)], and/ or were not conducted systematically (27). Therefore, this is the first systematic RF review that incorporates various forms of CA and various types of psychopathology. Given that adolescence and young adulthood are characterized by a heightened risk for psychopathology (29), we focus our review specifically on those RFs that benefit mental health in young people.

### Rationale

This preregistered systematic review offers health care providers a comprehensive overview of RFs that improve resilience to psychopathology in young people after CA. The results of our review potentially advance personalized therapy plans (14, 16), as well as preventative and public health interventions aimed at young people with a history of CA. Finally, this review aids the development of novel resilience theories and may therefore enhance our understanding of the complex concept of resilience factors.

### Objective

We aimed to identify empirically-supported RFs that reduce the risk of psychopathology in young people subsequent to CA. We focused on social, emotional, cognitive and behavioral RFs, as these factors are amenable to modification, and can be targeted in therapeutic and preventative interventions (16, 20).

## Methods

### Protocol and registration

On the 30th of November 2016 we preregistered our review protocol (30) at http://www.crd.york.ac.uk/PROSPERO/display_record.php?ID=CRD42016051978, to enable the reader to compare the suggested with the eventually conducted reviewing procedure.

### Information sources and search strategy

We searched English, Dutch and German literature in Web of Science, PsycINFO, and Scopus (e.g., including MEDLINE), for all years until November 2016. Search terms, searched documents and database specific search strategies can be found in Table 1.

**Table 1:** Used search strategy for the databases: Web of Science, Scopus, and PsycINFO.

**SEARCH TERMS**	
**Search category: title, abstract, & keywords**	
	(resilien^*^ OR advers^*^)
AND	(child^*^ OR infan^*^ OR adolescen^*^ OR teen^*^ OR youth^*^ OR pediatr^*^ OR paediatr^*^)
AND	(“self harm^*^” OR ^*^suicid^*^ OR psychopatholog^*^ OR psycholog^*^ OR psychiatr^*^ OR emotion^*^ OR affect^*^ OR mental^*^ OR disorder^*^)
**Search category: title**	
AND	(resilien^*^ OR protect^*^ OR support^*^ OR adapt^*^ OR promot^*^ OR moderat^*^ OR mediat^*^ OR predict^*^)
AND	(advers^*^ OR “at risk” OR hardship^*^ OR loss^*^ OR “family discord” OR parent^*^ OR trauma^*^ OR traged^*^ OR “chronic^*^^*^stress^*^” OR “life ^*^stress^*^” OR abus^*^ OR maltreat^*^ OR mistreat^*^ OR assault^*^ OR violen^*^ OR molest^*^ OR neglect^*^)
**SEARCHED DOCUMENTS**	
Types^*a^	(in press) articles, proceedings, conference papers, editorial materials, and electronic collections
**DATABASE SPECIFIC STRATEGIES**	
Scopus	We searched the subject areas “Health Sciences” (covering MEDLINE) and 'Social Sciences & Humanities'
PsycINFO	We additionally utilized subject headings for the two superordinate concepts: 'resilience' and 'childhood adversity': (“Resilience (Psychological)” OR “Protective Factors” OR “Adaptability (Personality)” OR “Adjustment” OR “Coping Behavior” OR “Emotional Adjustment” OR “Adaptive Behavior”) AND (“At Risk Populations” OR “Risk Factors” OR “Dysfunctional Family” OR “Emotional Trauma” OR “Trauma” OR “Chronic Stress” OR “Emotional Abuse” OR “Child Neglect” OR “Verbal Abuse” OR “Child Abuse” OR “Sexual Abuse” OR “Physical Abuse” OR “Violence” OR “Domestic Violence” OR “Exposure to Violence” OR “Social Deprivation”).

*a *We included all of the mentioned document types available for the three databases*.

### Study selection

Duplicates were filtered out using the Mendeley reference manager. Three reviewers (AdG, HC, & JF) pilot-screened 300 titles and abstracts in November 2016. The remaining articles were screened by two of the three reviewers with an approximately equal number of articles per pair. All articles were screened based on the PI(C)OS concept (31): Population (P), intervention (I; i.e., RF), outcome (O), and study design (S). When P, I, and O were met and the design was unknown, the full-text articles were screened for design. Incongruent ratings were solved through discussion, if necessary including a third author (PW).

### Study selection screening: eligibility criteria I

#### CA

CA, prior to age 18, was defined as one or multiple adversities (1, 2, 32), including: Loss of a significant other, discord within the family, poor parenting, traumatic life events/tragedy, chronic or life stress, hardship, at-risk environment, childhood abuse/maltreatment/mistreatment, and/ or childhood neglect. As we expect financial adversity to be indirectly related to psychopathology, via emotional adversity, we did not include financial adversity as CA (33, 34).

#### RFs

Inclusion criteria: The RF (a) is a direct effect, moderator, and/or a mediator for the relationship between CA and psychopathology, (b) belongs either to the individual-, family-, or community-level category, and (c) belongs to the cognitive, behavioral, social, and/or emotional functioning domain. Exclusion criteria: The RF is defined (a) as financial advantage, (b) as no re-victimization, (c) as inverse of CA, (d) as inverse of psychopathology, or is (e) not amenable.

#### Psychopathology

Psychopathology was defined as general mental distress, as self-harm behavior, as suicidal ideation, or as categorical diagnosis or continuous symptoms of any disorder included in the Diagnostic and Statistical Manual of Mental Disorders IV Text Revision [DSM-IV-TR, (35)].

#### Design

We included all longitudinal studies in which the RF was assessed before psychopathology, and CA was measured no later than the RF (i.e., cohort designs). Additionally, we excluded experimental designs which involved intervention on the RF.

### Study selection rescreening: eligibility criteria II

**Figure 1:**
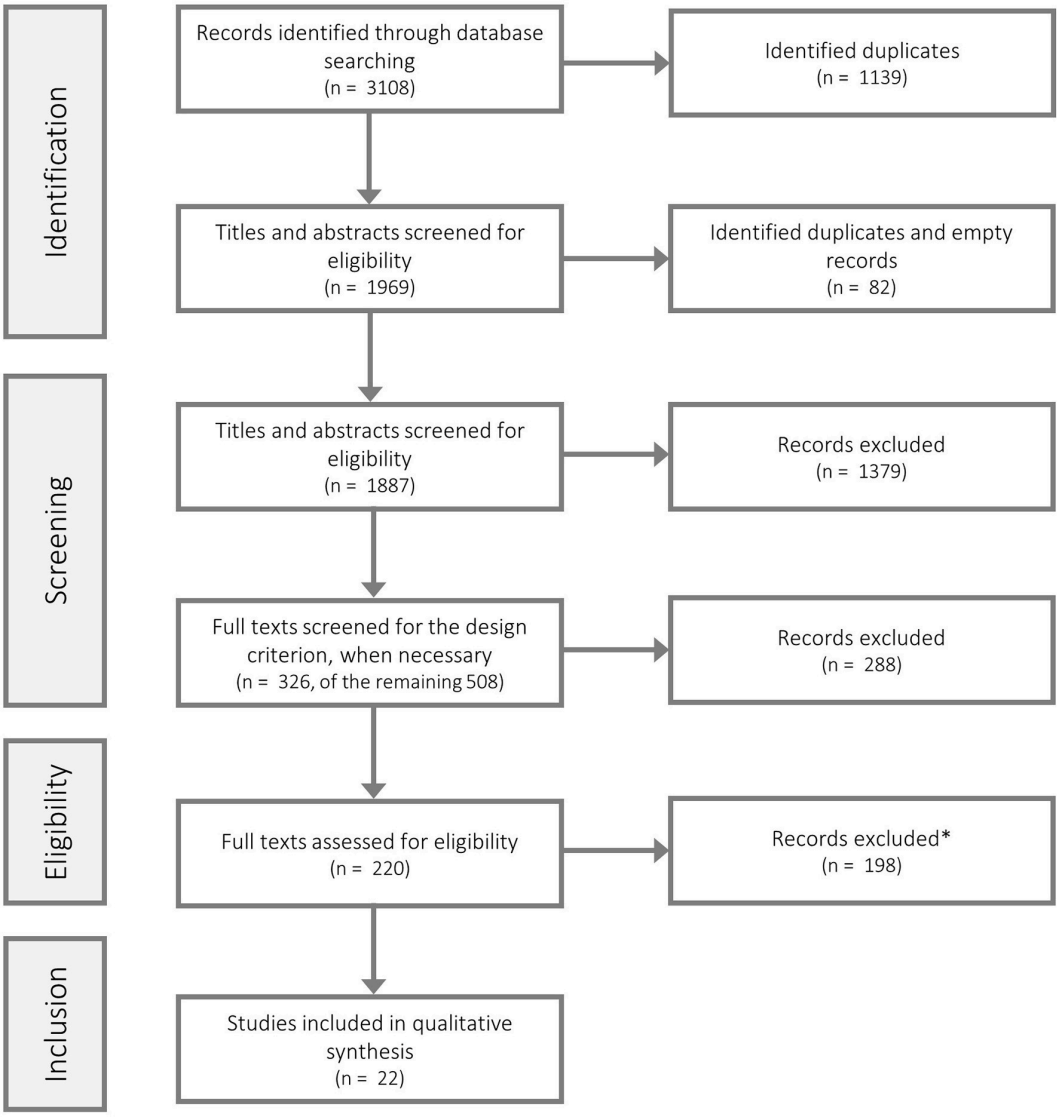
Study selection flow chart. We identified 878 potentially eligible studies in Web of Science, 1050 in Scopus and 1180 in PsycINFO. ^*^Of the 198 excluded articles of the eligibility review stage, one study was identified as duplicate and three studies were excluded due to insufficient information. The flow chart was modelled along the PRISMA recommendations (being under a Creative Commons Attribution License; see e.g. Liberati et al. (31), PLoS Med, can be retrieved from: https://doi.org/10.1371/journal.pmed.1000100).

The first screening led to more than 200 eligible articles. Therefore, we applied two additional selection criteria outlined below. AdG and JF rescreened the eligible articles in full-text, including the two additional selection criteria (see Figure 1; eligibility stage), which reduced the number of studies to a manageable number of 22 studies.

#### RFs

RFs should operate as moderator and/or mediator for the relationship between CA and psychopathology, as this indicates that the RF is specific to CA. When the RF is a direct effect, the RF may not be specific to CA and may operate the same for the whole population. We believe that this criterion is crucial, as it ensures that our “resilience factor” definition precisely matches our “resilience” definition, i.e., good mental health despite a history of adversity. In the case of mediation, if CA predicts a potential RF *positively* (e.g., high rumination), then a high level of this potential RF would have to predict psychopathology *positively* (e.g., high rumination leads to higher psychopathology). This means that a *low* level of this factor (e.g., low rumination) would be referred to as RF. Similarly, if CA predicts a potential RF *negatively* (e.g., low cognitive reappraisal), then a high level of this potential RF would have to predict psychopathology *negatively* (e.g., high cognitive reappraisal leads to lower psychopathology). Hence, a *high* level of this factor (e.g., high cognitive reappraisal) would then be referred to as RF. Thus, especially for adolescents with CA it would be advantageous to *reduce* the levels of *low* RFs (e.g., rumination) and to *enhance* the levels of *high* RFs (e.g., cognitive reappraisal), to subsequently lower psychopathology levels. In the case of moderation, lower levels of *low* and higher levels of *high* RFs reduce psychopathology levels more in adolescents with CA, compared to adolescents without CA. Hence, according to this criterion all RFs are especially crucial for adolescents with a history of CA.

#### Psychopathology

Psychopathology had to be assessed at a mean age of 13–24 years. This criterion is important to enable the systematic selection of more homogeneous studies, to ease and enhance the comparability of findings across studies. We chose this age range, because it is characterized by a heightened risk for psychopathology and thus allows for relevant and insightful conclusions (29).

### Mediation effects

The “eligibility criteria II” state that the RF must function as moderator and/or mediator for the relationship between CA and psychopathology. Yet, when referring to mediation effect we mean “positive mediation” effects, as “negative mediation” effects do not function as RFs. More specifically, when we refer to RFs that have been supported by mediation analyses, we exclusively refer to factors that operated as “positive mediators” —i.e., their relationships with both CA and psychopathology are in the same direction (i.e., either both are negative, or both are positive, as described in section RFs). A “negative mediator” would have opposite relationship directionalities with CA and psychopathology (i.e., one positive and one negative relationship), and therefore cannot function as an RF. Moreover, when we refer to a supported mediation effect, we expect that the association between CA and psychopathology is not significantly negative, as the mediator otherwise can also not function as an RF.

### Data extraction and quality assessment

The data extraction form was based on the STROBE report (36) and an adapted version of Downs and Black's (37) validated scale was used for the study quality ratings (see item templates in Supplement 2 and 3). AdG and JF conducted the data extraction pilot (3 studies: *M* Byrt's kappa = 0.56, *SD* = 0.29, range: 0.29–0.86; see Supplement 1A), the final data extraction (*M* Byrt's kappa = 0.74, *SD* = 0.17, range: 0.43–0.96; see Supplement 1A), and the study quality ratings (*M* Byrt's kappa = 0.61, *SD* = 0.19, range: 0.30–1.00; see Supplement 1A). Incongruent ratings were solved through consensus, if necessary including a third author (PW). When articles lacked relevant information, we emailed the corresponding authors. Moreover, to be able to systematically judge the quality of the reviewed moderation and mediation analyses, PW and JF additionally applied quality criteria to the analysis methods (i.e., adequacy of sample size, single vs. multiple RF model, quality of moderation/mediation analysis; see Supplement 4). Incongruent ratings were solved through consensus. Notably, the ratings of the analysis methods were not part of the pre-registered protocol and should therefore be considered as *post hoc* evaluation.

### Data synthesis method

Given that we conjectured to find a heterogeneous set of eligible studies (i.e., in terms of CA, RFs, and psychopathology) a quantitative meta-analysis would not be appropriate. Therefore, a narrative synthesis was conducted.

### Narrative description of moderating and mediating RFs

We shall describe moderation effects as follows: “the association between CA and psychopathology is weaker for adolescents with a higher (or lower) level of the RF.” We shall describe positive mediation effects as “a *high level of x* mediates the effect between CA and PP.” This means that a high level of CA is associated with a high level of x and a high level of x is in turn associated with a high level of psychopathology. Hence, a *low level of x* is the RF. On the other hand, if a *low level of x* mediates the effect between CA and PP, a *high level of x* is the RF (as a high level of CA is associated with a low level of x and a low level of x is in turn associated with a high level of psychopathology).

## Results

### Study selection

After electronically removing duplicates (1139 of the initial 3108 studies, see Figure 1), all 1969 remaining studies were screened based on title and abstract screening, according to the criteria of the study selection *screening* stage (Eligibility Criteria I). Of the 1969 studies we identified 82 as additional duplicates or empty records (which have not been identified electronically), resulting in 1887 potential studies. Of those 1887 studies 1379 did not meet the screening criteria (Eligibility Criteria I). The exclusion of these 1379 studies, resulted in 508 remaining potential studies. Of those 508 studies 182 met the eligibility criteria of stage 1. Yet, the remaining 326 studies (508–182) had to be screened in full-text, as for those studies we could not assess the design criterion only based on the title and the abstract. Of those 326 we could exclude 288 studies, resulting in 38 potentially eligible studies. Therefore, after initial screening we revealed 182 (508–326) potential studies which did not have to be screened in full text for the design criterion, plus 38 (326–288) potential studies that had to be screened in full text for the design criterion, resulting in total in 220 potentially eligible studies. Accordingly, those 220 studies were then rescreened in full text according to both the criteria of the study selection *screening* (Eligibility Criteria I) and the study selection *rescreening* (Eligibility Criteria II) stages. Of those 220, 198 studies could be excluded and 22 studies were thus eligible for data abstraction (Table 2).

**Table 2:** Methodological and sample characteristics of the analyzed studies.

**S**	**Gender**	**Analysis*N***	M^*a^age	**T for gender**	**% male**	**SES level**	**Nationality**	CA^*b^	**CAmeasure**	RF^*b^	**RFmeasure**	PP^*b^	**PPmeasure**
(38)	Both	244	12	T2 (baseline)	54.5	-	US	Emotional abuse	Quest.	Distress tolerance	Task	Anxiety symptoms	Quest.
(39)	Both	1973	14	T1	32	High	Australia	Adverse life experiences	Quest.	Expressive Suppression Cognitive reappraisal Rumination	Quest. Quest. Quest.	Psychological distress	Quest.
(40)	Both	451	–	T1	47.67	–	US	Marital distress/conflict	Quest. + task^*c^	Positive parenting	Quest. + task^*c^	Poor emotional well-being Externalizing Internalizing	Quest. Quest. Quest.
(41)	Both	59	17	T1	39	–	US	Physical, sexual abuse	Quest. + interview	Behav. reward reactivity Emotion. reward reactivity	Task Task^*e^	Depressive symptoms	Interview
(42)	Both	1501	-	T1	49.24	–	Palestine & Israel	Ethnic-political conflict	Quest.	Academic grades Self-esteem Positive parenting	Interview Interview Interview	PTS symptoms	Interview
(43)	Both	492	16	T1	47.5	–	US	Parental problem drinking	Quest.	Family cohesion Adolescent-mother com. Adolescent-father com.	Quest. Quest. Quest.	Externalizing	Quest.
(44)	Both	163	12	T2	50	–	Australia	Aggressive parenting	Task	Rumination	Quest.	Depressive symptoms	Quest.
(45)	Both	652	19	T2	32.2	–	–	Emotional, sexual, physical abuse	Quest.	Negative cognitive style Insecure attachment	Quest. Quest.	Depression symptomsAnxiety symptoms	Quest. Quest.
(46)	Both	312	14	i-sample	50	Low	US	Community violence	Quest.	Extended family support Parental involvement	Quest. Quest.	InternalizingExternalizing	Quest. Quest.
(47)	Both	6780	-	T1	42.2	–	Canada	Sexual abuse	Quest.	Maternal support	Quest.	Mental health problems	Quest.
(48)	Both	1064	-	T1	69	–	US	Parental violence	Quest.	Coping expectancy Enhancement expectancy	Quest. Quest.	Peak alcohol use Heavy episodic drinking	Quest. Quest.
(49)	Both	1643	14	T1 (i-sample)	49.4	Medium	Germany	Parental mentalhealth problems	Quest.	Self-efficacy Family climate Social support	Quest. Quest. Quest.	Depressive symptoms	Quest.
(50)	Both	585	-	T1	52	-	US	Physical abuse	Interview	Proactive parenting	Interview	Internalizing Externalizing	Quest. Quest.
(51)	Both	400	-	T1	59.25	Low	US	Emotional, sexual, physical abuse, neglect	Objective	Ego under vs. over-control Over-control vs. resilient Under-control vs. resilient	Quest.^*f^ Quest.^*f^ Quest.^*f^	Subtance use: cannabis Subtance use: alcohol Internalizing Externalizing	Interview Interview Quest. Quest.
(52)	Both	83	11	T2	48.8	Low	Palestine	Ethnic-political conflict	Quest.	Mental flexibility	Task	Emotional disorders^*g^ Emotional disorders^*h^ PTS symptoms	Quest. Quest. Interview
(53)	Both	332	-	T1 (i-sample)	45	-	Israel	Ethnic-political conflict (i.e., rocket attacks)	Quest.	School personnel support Friend support Immediate family support	Quest. Quest. Quest.	Violence commission Anxiety symptoms Depressive symptoms	Quest. Quest. Quest.
(24)	Both	771	-	i-sample	41.8	High	UK	Accumulated family adversity	Interview	Immediate family support Friendship support	Quest. Quest.	Depressive symptoms	Quest.
(54)	Female	360	22	T1	0	Low	US	Emotional, sexual, physical abuse	Quest.	Protective self-cognitions^*d^	Quest.	PTS symptoms	Interview
(55)	Both	189	-	i-sample	43.4	Medium	US	Adverse life experiences	Quest. + interview	Parenting quality	Quest.^*f^	Conduct	Quest. + interview
(56)	Both	1052	14	i-sample	52.6	Medium	Spain	Emotional abuse	Quest.	Disconnection/ rejection Impaired autonomy Other-directedness	Quest. Quest. Quest.	Social anxiety symptoms Depressive symptoms	Quest. Quest.
(57)	Both	2021	12	T1	49	-	US	Stressful family-level life events	Interview	Socialization Boldness Prosocial peers Academic engagement Parent-child relationship Antisocial peers	Quest. Quest. Quest. Quest. Quest. Quest.	Substance abuse	Interview
(58)	Both	2013	-	i-sample	52.4	-	Korea	Emotional, physical abuse, emotional, physical neglect	Quest.	Aggression	Quest.	Violent delinquency Non-violent delinquency	Quest. Quest.

CA, childhood adversity; RF, resilience enhancing factor; PP, psychopathology; S, study; T, assessment time point; i-sample, investigated sample; quest, questionnaire; behav., behavioral; emotion., emotional; com., communication; PTS, posttraumatic stress;

*a for CA assessment;

*b information regarding the assessment instruments can be requested from the author;

*c observer ratings on task performance;

*d this study contained interventions, but given that we did not expect the interventions to have an effect on the RF, we included the study;

*e subjective ratings integrated in task;

*f questionnaires completed by counselors/ interviewers;

*g self report;

*h *multiple reporters*.

### Study characteristics

**Figure 2:**
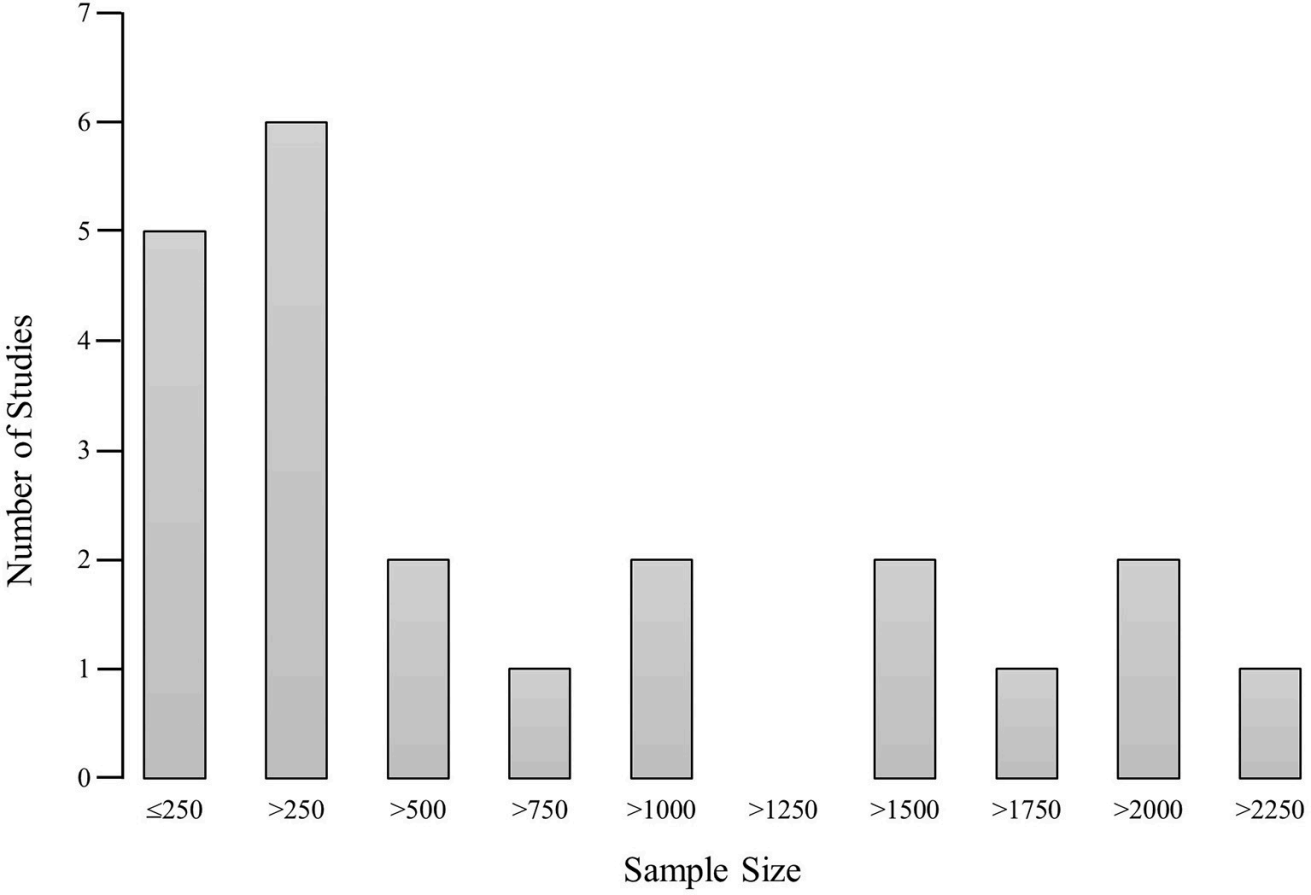
Sample size histogram. The histogram depicts the frequency of the studied sample sizes. The x-axis indicates the size of the studied sample in steps of 250 participants. The y-axis indicates the frequency of studies that investigated the belonging sample size.

All 22 studies were published in English, which is representative as only a negligible number of the screened articles were written in German or Dutch. Twenty-one of the studies included both genders (*M* male = 47.95%, *SD* = 8.27, range: 32–69%; see Supplement 1B). Walter et al. (54) included females only. The studies had a mean of 3.41 time points (*SD* = 1.65, range: 2–9), with a time frame ranging from 10 weeks to 16 years (*M* years = 4.55, *SD* = 4.37; see Supplement 1B). Sample sizes ranged from 59 to 6780 participants (*M* = 1052, *SD* = 1436; see Supplement 1B). As shown in Figure 2, 27.27% of the studies investigated more than 1,500 participants, 9.09% more than 1,000 participants, 13.64 percent more than 500 participants, and 50% fewer than 500 participants. Importantly, one of the 13 studies that conducted moderation analyses had a sample size below 77, which may be insufficient in terms of power. We used a sample size of 77 as guideline, as this is the sample size that is required for moderation analyses to detect a moderate effect (*f*^2^ = 0.15, power = 0.80, α = 0.05; see Supplement 5). However, all 12 studies that performed mediation analyses had sample sizes higher than 150, which we assume to be sufficient in terms of power. We used a sample size of 150 as guideline, as MacKinnon, Fairchild and Fritz (59) report that a sample size of 100 to 200 was sufficient even for multiple mediator models. At the CA assessment, the participants' mean age was 14.75 years (*SD* = 3.25, range: 11–22; see Supplement 1B). Four studies utilized a low, three a medium and two a high socio-economic status (SES) sample. Thirteen studies lacked information or did not provide an interpretation for SES. Twelve studies were performed in the United States or Canada, three in Europe, three in Israel and/ or Palestine, two in Australia, one in Korea, and one lacked information.

In total, 15 types of CAs were assessed (Supplement 6): Five types of childhood maltreatment (nine studies), seven types of intra-family adversity (seven studies), two types of community adversity (four studies) and one clustered type of adverse life experiences (two studies). Moreover, five types of disorders and four clustered types of psychopathology have been assessed (Supplement 6), with a mean of 1.59 assessed types of psychopathology per study (*SD* = 0.80, range: 1–3). Overall, 46 RFs were examined (Table 3), with a mean of 2.09 RFs per study (*SD* = 1.23, range: 1–6).

**Table 3:** Resilience factors tested in the analyzed studies, split into individual, family, and community level.

**Individual**		**Family**		**Community**	
**Supported**	**Not supported**	**Supported**	**Not supported**	**Supported**	**Not supported**
High distress tolerance [MO (38)]	–	High positive parenting [ME (40)]+[MO (42)]	Positive parenting [MO (40)]	High social support [MO (49)]^*a^	**-**
High cognitive reappraisal (MO + ME (39)]	-	High family cohesion [ME (43)]	Family cohesion [ME (43)]	**-**	Friend support [MO (53)]+[ME (24)]
Low expressive suppression [ME (39)]	Expressive suppression [MO (39)]	–	Adolescent-father communication [ME (43)]	–	School support [MO (53)]
Low rumination [ME (39)]	Rumination [MO (39)]+[ME (44)]	–	Adolescent-mother communication [ME (43)]	–	Prosocial peers [ME (57)]^*c^
–	Behavioral reward reactivity [MO (41)]	High extended family support [MO (46)]	Extended family support [MO (46)]	–	Antisocial peers [ME (57)]^*c^
–	Emotional reward reactivity [MO (41)]	High parental involvement [MO (46)]	Parental involvement [MO (46)]		
–	Academic grades [MO (42)]	**-**	Maternal support [MO (47)]		
High self-esteem [MO (42)]	-	Positive family climate [MO (49)]	-		
Low insecure attachment [ME (45)]	Insecure attachment [ME (45)]	-	Proactive parenting [MO (50)]		
-	Negative cognitive style [ME (45)]	High immediate family support [MO (53)]+[ME (24)]	Immediate family support [MO (24, 53)]		
Low coping expectancy^def1^ [ME (48)]	-	-	Parenting quality [MO (55)]^*b^		
-	Enhancement expectancy^def2^ [ME (48)]	-	Parent-child relationship [ME (57)]^*c^		
-	Self-efficacy [MO (49)]				
Low ego over-control [ME (51)]	Ego over-control [ME (51)]				
Low ego under-control [ME (51)]	Ego under-control [ME (51)]				
-	Ego under- vs. over-control [ME (51)]				
High mental flexibility [MO (52)]	Mental flexibility [MO (52)]				
-	Protective self-cognitions [ME (54)]				
Low disconnection/rejection [ME (56)]^*c^	Disconnection/rejection [ME (56)]^*c^				
Low other-directedness [ME (56)]^*c^	Other-directedness [ME (56)]^*c^				
-	Impaired autonomy [ME (56)]^*c^				
-	Socialization [ME (57)]^*c^				
-	Boldness [ME (57)]^*c^				
-	Academic engagement [ME (57)]^*c^				
Low aggression [ME (58)]^*c^	Aggression [ME (58)]^*c^				

*MO, moderation analysis; ME, mediation analysis*.

*a *The social support measure could potentially also include family support and should therefore also belong to the family domain*.

*b *The CA timeline requirements might not be fully met*.

*c *The analysis did not include the direct path between CA and psychopathology when calculating the indirect mediation effect of the RF*.

def1 Definition, Consuming alcohol to handle stress;

def2 *Definition, Consuming alcohol to improve mood*.

### Individual-level RFs

We report findings of individual-level RFs (Table 3) within four clusters. In total we found 13 supported individual-level RFs including three cognitive, four emotion regulation, three social interaction/attachment and three personality/self-concept RFs:

#### Cognition and academic performance

Qouta et al. (52) found that the positive relationship between traumatic events (i.e., ethnic-political conflict) and emotional disorders (i.e., internalizing and externalizing symptoms) was stronger for adolescents with lower levels of mental flexibility (moderation). Yet, mental flexibility did not moderate the relationship between traumatic events and posttraumatic stress symptoms (52). In the study of Boyes et al. (39) the association between a history of adverse life events and psychological distress was weaker for adolescents who reported more cognitive reappraisal (moderation). Low cognitive reappraisal also mediated the association between a history of adverse life events and psychological distress (39). Similarly, Boyes et al. (39) revealed that high rumination mediates the association between a history of adverse life events and psychological distress. However, no moderation effect was found for rumination (39). Gaté et al. (44) found that rumination does not mediate the association between aggressive parenting and depressive symptoms. Moreover, Hankin (45) reported that a negative cognitive style no longer mediates the relationship between emotional abuse and subsequent depressive symptoms, when controlling for negative life events and an insecure attachment style. Hankin (45) did not investigate mediation effects for other combinations of CA (i.e., sexual, physical, and/or emotional abuse) and psychopathology (depressive or anxiety symptoms), as pairwise associations between variables were lacking. For the same reason, Hicks et al. (57) did not analyse the mediation effect of academic engagement along the relationship between stressful life events and substance abuse. Finally, Dubow et al. (42) found that academic grades do not moderate the association between ethnic-political conflict (e.g., violence) and posttraumatic stress symptoms. In sum, we found support for high mental flexibility, high cognitive reappraisal, and low rumination as RFs.

#### Emotion regulation

Banducci et al. (38) found that adolescents with less distress tolerance and more emotional abuse experienced the most anxiety symptoms in the long term (moderation). Along these lines, Boyes et al. (39) revealed that high expressive suppression mediates the association between a history of adverse life events and psychological distress, however, no moderation effect was found. In the study of You and Lim (58), high aggression mediated the association between abuse (emotional and physical) and violent as well as non-violent delinquency. High aggression also mediated the association between emotional neglect and violent delinquency, as well as between physical neglect and non-violent delinquency. However, aggression did not mediate the association between emotional neglect and non-violent delinquency, as well as between physical neglect and violent delinquency (58). Jester et al. (48) showed that high alcohol coping expectancy, i.e., consuming alcohol to handle stress, mediates the association between inter-parent violence and both peak alcohol use and heavy episodic drinking (when taking distress as intermediate predictor into account). In contrast, no mediation effects were found for alcohol enhancement expectancy, i.e., consuming alcohol to improve mood (48). Finally, Dennison et al. (41) found that emotional and behavioral reward reactivity did not moderate the relationship between childhood maltreatment (physical and/or sexual abuse) and subsequent depressive symptoms. Hence, high distress tolerance, low expressive suppression, low aggression, and low alcohol coping expectancy were supported as RFs.

#### Attachment and social interactions

Hankin (45) found that high insecure attachment mediates the relationship between emotional abuse and depressive symptoms. No mediation effects were analyzed for other combinations of CA (i.e., sexual, physical, and/ or emotional abuse) and psychopathology (i.e., depressive or anxiety symptoms), due to the lack of pairwise associations (45). Calvete (56) investigated disconnection/rejection, other-directedness and impaired autonomy factors along the relationship between two CAs (i.e., abuse by parents and peers) and two psychopathology variables (i.e., depressive and social anxiety symptoms). High disconnection/rejection mediated the relationship between abuse by peers and depressive symptoms. High other-directedness mediated the relationship between abuse by peers and social anxiety. Due to the absence of pairwise associations, no mediation effects were analyzed for other combinations of CA and psychopathology, or for impaired autonomy (56). Finally, Hicks et al. (57) found that socialization (e.g., obeying rules and committing to ethical values) does not mediate the relationship between stressful life events and substance abuse. Additionally, due to the absence of pairwise associations, no mediation effect was analyzed for boldness [e.g., social confidence, adaptability to distress, and sensation seeking (57)]. Therefore, low insecure attachment, low disconnection/rejection and low other-directedness were supported as RFs.

#### Personality and self-concept

Oshri et al. (51) studied the putative RF ego control, which was split into: (a) ego over-control vs. ego resilience, (b) ego under-control vs. ego resilience and (c) ego under-control vs. ego over-control. High ego over-control vs. resilience mediated the association between early child maltreatment and internalizing, but not between early child maltreatment and cannabis use, alcohol use (see Supplement 1C), or externalizing behaviors. High ego under-control vs. resilience mediated the association between early child maltreatment and cannabis use, internalizing and externalizing behaviors, but not between early child maltreatment and alcohol use. For ego under-control vs. ego over-control no mediation effects were found (51). Dubow et al. (42) found that the association between ethnic-political conflict (e.g., violence) and posttraumatic stress symptoms was only significant for adolescents with a low amount of self-esteem (moderation). In contrast, in the study of Klasen et al. (49) self-efficacy did not moderate the association between parental psychopathological problems and the development of depressive symptoms in the adolescent offspring. Similarly, in the study of Walter et al. (54), protective self-cognitions (i.e., self-esteem and self-efficacy) did not mediate the association between child abuse and posttraumatic stress symptoms (taking resource loss as intermediate mediator into account). Thus, in the personality/self-concept cluster we found support for low ego over-control, low ego under-control, and high self-esteem.

### Family RFs

We split family-level RFs (Table 3) into two clusters and found empirical support for four family support and two parenting RFs:

#### Family support

Hardaway et al. (46) found that the effect of community violence on externalizing behaviors was only significant for adolescents with a small amount of extended family support (moderation). No effect was found for the relationship between community violence and internalizing behaviors (46). Van Harmelen et al. (24) showed that low immediate family support mediates the relationship between accumulated family adversity and depressive symptoms. No moderation effect was found (24). Similarly, Shahar and Henrich (53) revealed that immediate family support significantly attenuates the relationship between exposure to rocket attacks and both subsequent depressive symptoms and severe commission of violence (moderation). Yet, immediate family support did not moderate the relationship between exposure to rocket attacks and anxiety (53). Moreover, Finan et al. (43) found that low family cohesion mediates the association between paternal alcohol abuse problems and both violation of rules (boys and girls) and aggressive conduct (girls only). No mediation effect was found for any other combination of CA (i.e., maternal or paternal alcohol abuse problems) and psychopathology [i.e., alcohol use, drug use, or binge drinking (43)]. Similarly, in the study of Klasen et al. (49), the positive relationship between parental psychopathological problems and the development of depressive symptoms in the adolescent offspring was mitigated for adolescents who experienced a better family climate (moderation). Hence, we found support for high extended family support, immediate family support, family cohesion, and a positive family climate as RFs.

#### Parental support

Hardaway et al. (46) found that the effect of community violence on externalizing behaviors was only significant for adolescents with a small amount of parental involvement (moderation). Yet, parental involvement did not moderate the relationship between community violence and internalizing behaviors (46). Similarly, Dubow et al. (42) found that the association between ethnic-political conflict (e.g., violence) and posttraumatic stress symptoms was only significant for adolescents with a low amount of positive parenting (moderation). Cui and Conger (40) found that low positive parenting (i.e., high positive parenting includes low negative parenting) mediates the association between marital problems and poor emotional well-being, internalizing, as well as externalizing symptoms. Moderation effects for positive parenting were mostly not supported, as only one out of 12 effects was significant [i.e., for the association between marital distress and poor emotional well-being (40)]. Due to the absence of direct associations, Hicks et al. (57) did not analyse the mediation effect of the parent-child relationship for the association between stressful life events and substance abuse. Moreover, in the study of Masten et al. (55), parenting quality did not moderate the association between adverse life experiences and conduct symptoms. Similarly, Lansford et al. (50) found that proactive parenting does not moderate the relationship between physical abuse and change in both internalizing symptoms and externalizing behaviors.

Two studies focussed on RFs specific to one parent (43, 47). Finan et al. (43) found that adolescent-mother and adolescent-father communication (see Supplement 1D) do not mediate the association between parental alcohol abuse problems (i.e., maternal and paternal) and externalizing indicators (i.e., alcohol use, drug use, violation of rules, aggressive conduct, and binge drinking). Likewise, Hébert et al. (47) found that maternal support does not moderate the association between childhood sexual abuse and mental health problems. Thus, in sum, parental involvement and positive parenting were supported as RFs.

### Community RFs

On the community level, Klasen et al. (49) found that the positive association between parental psychopathological problems and the development of depressive symptoms in the adolescent offspring is mitigated for adolescents who experienced more social support (moderation). In contrast, in the study of Shahar and Henrich (53) school and friendship support did not moderate the relationship between exposure to rocket attacks and depressive symptoms, anxiety symptoms, as well as severe violence commission. Due to the absence of pairwise associations, van Harmelen et al. (24) did not investigate the mediation effect of friendship support for the relationship between accumulated family adversity and depressive symptoms. For the same reason, Hicks et al. (57) did not analyse the mediation effects of prosocial and antisocial peers along the relationship between stressful life events and substance abuse. Therefore, on the community-level high social support was supported as RF.

### Single vs. multiple RFs

Of the 22 studies, only eight have tested indirect (i.e., mediation) and/ or interaction (i.e., moderation) effects, while correcting for at least one other RF. Calvete [other-directedness, disconnection/rejection, impaired autonomy (56)], Finan et al. [family cohesion, adolescent-mother communication, adolescent-father communication (43)], Hankin [insecure attachment, negative cognitive style (45)], Hicks et al. [socialization, boldness, prosocial peers, antisocial peers, academic engagement, parent-child relationship (57)], as well as Jester et al. [alcohol coping expectancy, alcohol enhancement expectancy (48)] tested mediation effects, while correcting for at least one other RF. Dubow [self-esteem, positive parenting, academic grades (42)] as well as Shahar and Henrich [immediate family support, school personnel support, friend support (53)] tested interaction effects in models containing more than one RF interaction. Boyes et al. (39) tested the indirect as well as the interaction effects of three RFs (expressive suppression, cognitive reappraisal, rumination). Yet, while the mediation analysis was corrected for the respective other two RFs, in the moderation model two RFs were only entered as main effects, not as interactions [expressive suppression, rumination (39)]. Hence, the current literature contains some effort to establish complex RF models that test mediation and moderation effects of RFs, while controlling for the impact of other RFs.

None of the eight mentioned studies included a model with more than six RFs. Jester et al. (48) as well as Hankin (45) first tested the indirect RF effects separately, before they performed a multiple RF model correcting for the respective other RFs. Jester et al. (48) showed that alcohol coping expectancy was a significant mediator in the single and the multiple RF model, whereas alcohol enhancement expectancy was neither significant in the multiple nor in the single RF model. In contrast, in Hankin's (45) study insecure attachment was a significant mediator in the single and the multiple RF model, whereas negative cognitive style was only a significant mediator in the single RF model. Hence, controlling for the interrelation between RFs is important as some RFs may only be significant when being tested in isolation, but not when being tested simultaneously with other individual, family, and community RFs. Along these lines, three studies found support for more than one RF in multiple RF models. This finding supports the notion that not one RF in isolation but complex interrelations of RFs affect the relationship between CA and psychopathology. In sum, such findings strongly underpin the need for a complex model that can account for various RFs following adversity, when predicting psychopathology.

### Quantifying RF effects

Comparing the effects of moderating and mediating effects statistically was not possible, as the reviewed RFs were studied following as many as 15 different forms of adversities, in the attempt to predict as many as five types of disorders (anxiety symptoms, depressive symptoms, posttraumatic stress symptoms, substance (ab)use symptoms, and conduct symptoms) and four clustered types of psychopathology (psychological distress, mental well-being, externalizing, and internalizing). Given such a variety of studied contexts, we believe that statistical comparison is not feasible. Some studies did report model related fit indices {moderation: e.g., *R*^2^ (49, 52); mediation: e.g., Root Mean Square Error of Approximation [RMSEA; e.g. (24, 40, 54, 58)]} but the majority of the studies did not report RF related effect sizes. The manual calculation of the effect sizes for mediating RFs might theoretically have been possible, as the proportion mediated (indirect effect divided through the total effect) could be calculated (59). Yet, the interpretation of the proportion mediated is conditional on the total effect (i.e., a small proportion mediated of a large total mediation effect might with regard to actual effect still be strong, while a large proportion mediated of a small total mediation effect might with regard to the actual effect still be weak). Given that the total effects of the studies, being based on 15 different independent adversity variables and nine different dependent psychopathology variables, are so numerous, the proportion mediated would not have been comparable between studies. Moreover, the proportion mediated is only robust for sample sizes of 500 or larger (59), which would only have been the case in seven studies (24, 39, 45, 48, 56–58), of which three are statistically controversial as they lack the impact of the direct effect (56–58). Similarly, we considered the calculation of effect sizes for moderation RFs as not feasible. Firstly, standard effect sizes such as the incremental R^2^, which indicates the contribution of an interaction to the moderation model, are difficult to interpret, as they merely designate the contribution of an interaction and not the magnitude of its effect (60). Moreover, for more advanced calculations of effect sizes the necessary information, such as the Mean Square Residuals [MSR (61)], was not provided.

### Study quality

#### Reporting, internal and external validity

**Figure 3:**
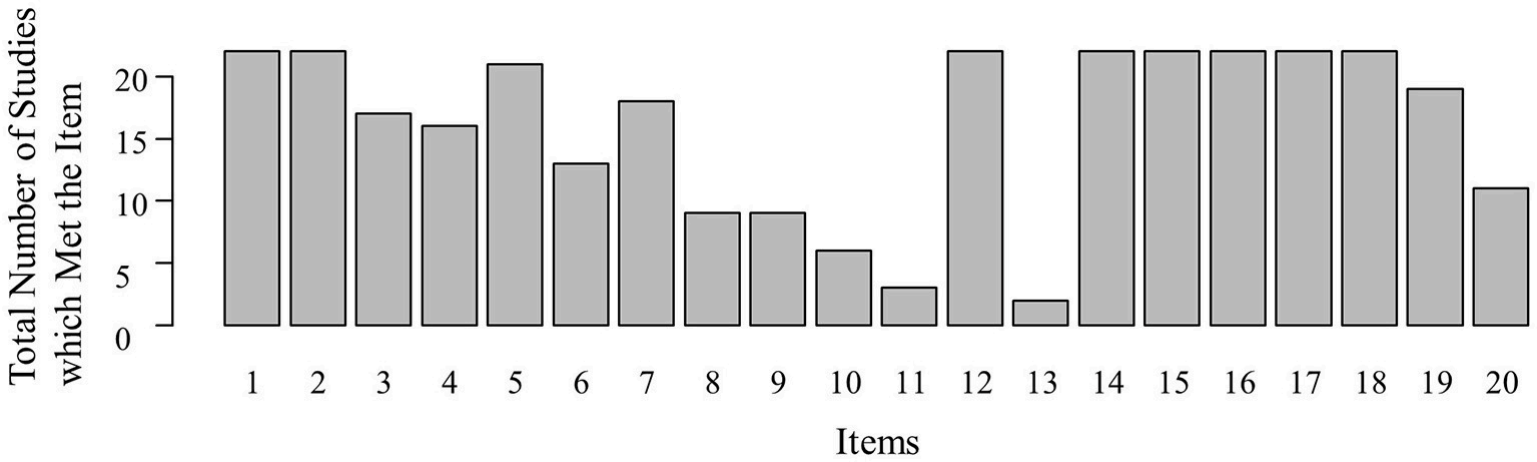
Quality rating distribution. The number of studies (y) which met the respective item of the adapted version of Downs' and Black's (37) quality rating scale (x). 1, Clarity of study aim; 2, Sufficient description of outcome(s); 3, Sufficient description of participant characteristics; 4, Presence of description of confounders; 5, Appropriate description of findings; 6, Report of variability estimates; 7, Description lost to follow-up characteristics; 8, Report of exact *p*-values; 9, Representativeness of recruitment cohort; 10, Representativeness of participation cohort; 11, Blinding; 12, Clarity about data dredging; 13, Adjustment for variability in follow-up length between participants; 14, Adequacy of statistical tests; 15, Accurate CA measure(s); 16, Accurate RF measure(s); 17, Accurate psychopathology measure(s); 18, Recruitment of same population for participants of different CA groups; 19, Correction for confounding; 20, Loss to follow-up correction.

Individual quality items were met by a mean of 16 studies (Figure 3; *SD* = 6.97, range: 2–22). The quality item “adjustment for variability in follow-up length between participants” [item 13 (37)] was the least frequently met item, being met by only two studies (41, 52). Similarly, the item assessing whether the researchers who measured psychopathology were in experimental terms blind (item 11), was only met by three studies (39, 51, 56). In contrast, as much as eight quality rating items (items 1, 2, 12, and 14–18) were met by all studies. Those eight included for example the items “clarity of study aim” (item 1) or “sufficient description of the psychopathology variable” (item 2). Overall, all studies met more than half of the assessed quality items. Therefore, we concluded that all studies were of sufficient quality to be included (*M* = 14.55, *SD* = 2.04, range: 11–18).

#### Quality of the analytic methods

Ten studies performed moderation [five multiple regression analyses (MRAs), three growth models, two path models (38, 41, 42, 46, 47, 49, 50, 52, 53, 55)], nine mediation [one MRA, seven path models or structural equation models (SEMs), one SEM based on probit regression (43–45, 48, 51, 54, 56–58)] and three both types of analyses [four MRAs, two SEMs (24, 39, 40)]. Three studies (56–58) did not control for the direct effect between CA and psychopathology when calculating mediation effects, which violates Baron and Kenny's (62) traditional mediation approach. Moreover, in Masten and colleagues' (55) study, parts of the CA index may have been assessed later than the RF. Hence, these four studies should be interpreted with caution.

To be able to judge the qualitative value of the moderation and mediation analyses we additionally applied quality criteria to the analysis methods (i.e., this was not part of the pre-registered protocol and should therefore be considered as *post hoc* evaluation). Moderation analyses received (a) a “1” when lacking correlational and significance testing for the relationship between CA and psychopathology at different levels of the moderator variable, (b) a “2” for correlational *post hoc* probing of the relationship between CA and psychopathology at different levels of the moderator variable, and (c) a “3” for regression analytic *post hoc* probing of the relationship between CA and psychopathology at different levels of the moderator variable. Detailed descriptions of these analytic methods can be found in Holmbeck (63). Mediation analyses received (a) a “1” for either no calculation of the overall indirect effect or the usage of the “direct effect reduction to non-significance” criterion, (b) a “2” for the calculation of the Sobel test or comparable indirect effect tests, and (c) a “3” for the usage of bootstrap methods for the calculation of the indirect effect. Detailed descriptions of these analytic methods can be found in MacKinnon et al. (59). The quality ratings can be found in Table 4.

**Table 4:** Quality ratings for the analysis methods that were used to analyse the resilience factors, split into individual, family, and community level.

**Resilience factor**	**Study**	**Moderation quality rating**	**Moderation supported**	**Mediation quality rating**	**Mediation supported**
**INDIVIDUAL LEVEL**					
Distress tolerance	(38)	3	Yes	NA	NA
Cognitive reappraisal	(39)	3	Yes	3	Yes
Expressive suppression	(39)	3	No	3	Yes
Rumination	(39)	3	No	3	Yes
Rumination	(44)	NA	NA	3	No
Behavioral reward reactivity	(41)	3	No	NA	NA
Emotional reward reactivity	(41)	3	No	NA	NA
Academic grades	(42)	3	No	NA	NA
Self-esteem	(42)	3	Yes	NA	NA
Insecure attachment	(45)	NA	NA	2	Yes
Negative cognitive style	(45)	NA	NA	2	No
Coping expectancy^def1^	(48)	NA	NA	2	Yes
Enhancement expectancy^def2^	(48)	NA	NA	2	No
Self-efficacy	(49)	1	No	NA	NA
Ego over-control	(51)	NA	NA	3	Yes
Ego under-control	(51)	NA	NA	3	Yes
Ego under- vs. over-control	(51)	NA	NA	3	No
Mental flexibility	(52)	1	Yes	NA	NA
Protective self-cognitions	(54)	NA	NA	1	No
Disconnection/rejection^*c^	(56)	NA	NA	3	Yes
Other-directedness^*c^	(56)	NA	NA	3	Yes
Impaired autonomy^*c^	(56)	NA	NA	3	No
Socialization^*c^	(57)	NA	NA	2	No
Boldness^*c^	(57)	NA	NA	2	No
Academic engagement^*c^	(57)	NA	NA	2	No
Aggression^*c^	(58)	NA	NA	3	Yes
**FAMILY LEVEL**					
Positive parenting	(40)	1	No	2	Yes
Positive parenting	(42)	3	Yes	NA	NA
Family cohesion	(43)	NA	NA	2	Yes
Adolescent-father communication	(43)	NA	NA	2	No
Adolescent-mother communication	(43)	NA	NA	2	No
Extended family support	(46)	3	Yes	NA	NA
Parental involvement	(46)	3	Yes	NA	NA
Maternal support	(47)	3	No	NA	NA
Positive family climate	(49)	1	Yes	NA	NA
Proactive parenting	(50)	3	No	NA	NA
Immediate family support	(53)	3	Yes	NA	NA
Immediate family support	(24)	Not rateable	No	2	Yes
Parenting quality^*b^	(55)	3	No	NA	NA
Parent-child relationship^*c^	(57)	NA	NA	2	No
**COMMUNITY LEVEL**					
Social support^*a^	(49)	1	Yes	NA	NA
Friend support	(53)	3	No	NA	NA
Friend support	(24)	NA	NA	2	No
School support	(53)	3	No	NA	NA
Prosocial peers^*c^	(57)	NA	NA	2	No
Antisocial peers^*c^	(57)	NA	NA	2	No

*NA, not performed; Not rateable, no significant effect and no information provided whether post hoc tests were applied in case of significant effects (i.e., in case of nonsignificant effects, follow up post hoc probing tests are not necessary for moderation)*.

*a *The social support measure could potentially also include family support and should therefore also belong to the family domain*.

*b *The CA timeline requirements might not be fully met*.

*c *The analysis did not include the direct path between CA and psychopathology when calculating the indirect mediation effect of the RF*.

def1 Definition, Consuming alcohol to handle stress;

def2 *Definition, Consuming alcohol to improve mood*.

Of the 13 studies which analyzed moderation effects, one study could not be rated for its analytic quality, as it did not contain a description of whether *post hoc* probing would have been performed in case of significant interaction effects (24). Moreover, three of the 12 studies were rated with a “1” (see Table 4) and the remaining nine studies with a “3.” Of the 12 studies that tested mediation, one study was rated with a “1,” six studies were rated with a “2,” and five studies were rated with a “3.” In sum, we concluded that the majority (moderation: 75%; mediation: 91.67%; total 83.34%) of the analytic methods that were used by the studies to test RFs are in line with the general guidelines for testing moderation and mediation, and can be considered as qualitatively adequate.

Splitting the results into systemic levels (i.e., individual, family, and community levels) showed that for the individual level RFs 80% of the moderation analyses and 94.74% of the mediation analyses were qualitatively adequate (rating of “2” or higher). For the family level RFs 77.78% of the moderation analyses and 100% of the mediation analyses were qualitatively adequate. Similarly, for the community level RFs 66.67% of the moderation analyses and 100% of the mediation analyses were qualitatively adequate. The analytic quality was examined in percentages to control for the impact of the differing number of performed analyses on each systemic level. Overall, we did not identify any trend regarding analytic quality differences between individual, family, and/ or community RFs.

## Discussion

The aim of this systematic review was to identify empirically supported RFs that benefit mental health in young people following CA. We reviewed 22 studies, including 46 amenable RFs. Thirteen of 25 individual-level RFs, six of 12 family-level RFs, and one of five community-level RFs were confirmed to significantly reduce the risk of psychopathology following CA. The absolute number of supported RFs seems to indicate that individual- and family-level RFs are most effective. However, the seemingly lower relevance of community-level RFs may be artefactual due to the small number of community-level studies that we could include in this review.

The 13 supported individual-level RFs included three cognitive (high: cognitive reappraisal, mental flexibility; low: rumination), four emotion regulation (high: distress tolerance; low: alcohol coping expectancy, aggression, expressive suppression), three social interaction/attachment (low: insecure attachment, disconnection/rejection, other-directedness) and three personality/self-concept RFs (high: self-esteem; low: ego over-control, ego under-control). It is as yet unknown whether these RF dimensions have compensatory effects, in the sense that an individual who performs low on one of those dimensions might still be functioning resiliently through performing high on other dimensions. Moreover, for most of the RFs it is also unknown to what extent they overlap in their prediction of mental health resilience.

Supported family-level RFs consisted of four family support (high: family cohesion, positive family climate, immediate family support, extended family support) and two parenting RFs (high: positive parenting, parental involvement). Interestingly, all RFs that were specific to one parent, e.g., adolescent father communication or maternal support, were not supported as RFs. This may suggest that the totality of family support is more important for resilience, than the quality of support from individual family members. Yet, as for the individual-level RFs, it is unknown to what extent the RFs overlap in their prediction of mental health resilience.

The fact that on the community-level only high social support was revealed as RF might suggest that a general social network has a stronger resilience enhancing effect than specific types of social support. However, given the restricted number of included community-level studies this conclusion is rather preliminary and requires further investigation. For example, our lab recently found that friendship support predicts resilient functioning in young people (64). Thus, although only one RF was revealed on the community-level, this does not suggest that community-level RFs are less important for mental health resilience. Rather, community-level RFs have had less attention than individual- and family-level RFs and therefore require further investigation. A more thorough examination of community-level RFs may enhance our understanding of the overall picture of systemic levels that benefit mental health resilience. On the whole, our review found support for RFs on all studied systemic levels, i.e., individual-, family- and community-levels, which indicates a movement toward a more complete understanding of the resilience concept.

Despite the movement to a more systemic approach, only eight of the reviewed studies corrected for the impact of at least one other RF, when testing the indirect and/or interaction effect of an RF (i.e., multiple RF model). Findings of single vs. multiple RF models indicated that taking the interrelatedness of RFs into account is important, as some RFs may only be significant when being tested in isolation, but not when being tested simultaneously with other individual, family, and/or community RFs. Along these lines, three studies found support for more than one RF in multiple RF models. This supports the notion that not one RF in isolation but complex interrelations of RFs affect the relationship between CA and psychopathology. Such findings strongly underpin the need for a complex model that can account for various RFs following adversity that benefit mental health resilience.

It would have been advantageous if effect sizes could have been calculated for moderation and mediation effects. This would have allowed us to draw conclusions regarding the magnitude of specific RF effects. Knowing the magnitude of RFs is beneficial, as it gives an indication about which factors might be most efficient when being approached in therapy. In the future, open data sharing, as was for example done by van Harmelen et al. (24), may facilitate RF comparisons. Given that our findings suggest that RFs do not function in isolation but in complex interrelated systems, it would be advantageous to know effect sizes of isolated RF effects, yet it would perhaps be even more interesting to establish and examine the effects of several RFs being clustered in complex systems of unidirectional or directional interrelations.

For a systematic review it is of critical importance to carefully assess and investigate the (a) reporting, (b) internal, (c) external, and (d) the analytic quality of the studies. As all studies met more than half of the assessed quality items (i.e., for reporting, internal, and external validity), we decided that all studies were of sufficient quality to be included. However, the quality ratings were not without limitations. For example, Downs and Black's (37) quality criteria are not specific to cohort studies and some more recent statistical improvements, such as the match of the variable level and the analysis technique (e.g., categorical vs. continuous data analysis methods), are not directly covered. Critics might further argue that the impact of studies in a systematic review should be weighed according to the study quality. Given that the set of reviewed studies was highly disparate and fairly incomparable, weighing according to “reporting,” “internal,” or “external” validity criteria would not have been insightful. Yet, as the systematic review focussed on moderating and mediating RFs, we considered it most insightful to apply weights based on the quality of the applied moderation and mediation methods. Of the studies that (a) performed moderation analysis and (b) could be rated for the analytic quality, 75% applied qualitatively adequate analysis techniques. Of the studies that tested mediation, 91.67% applied adequate analysis techniques. Therefore, we concluded that the majority (83.34%) of the applied analytic methods could be considered as qualitatively adequate. Moreover, we did not identify any trend regarding analytic quality differences between individual, family and/ or community RFs. We believe that this finding supports our conclusion that RFs are not restricted to one systemic level but are found to function on all three investigated systemic levels. Therefore, we call future research to focus on a more systemic and complete understanding of the RF concept.

The reviewed studies were conducted in as many as eight different countries: United States [11 studies], Israel and/or Palestine [3 studies], Australia [2 studies], Canada [1 study], UK [1 study], Spain [1 study], Germany [1 study], and in Korea [1 study]. Moreover, all 22 reviewed studies were published in English and only a negligible number of the 1969 screened studies were published in German and Dutch. Hence, research scrutinizing resilience promoting factors seems to be an international imperative. Yet it needs to be noted, that despite the variety of studied nations, mainly Western populations were studied.

Even though the studies were highly disparate, 95.45% of the studies researched both genders with on average 47.95% males per sample. Therefore, we consider the review overall as gender balanced and on average gender representative. Nine studies provided a proper SES description, which covered a range from low to high SES (4 low, 3 medium, 2 high). However, we believe that not enough studies have provided sufficient information to draw a conclusion regarding the studies' representativeness of SES. Along these lines, no conclusion can be drawn whether RFs operate the same for adolescents with different SES levels. Similarly, as the studies varied strongly in the studied time frame, which ranged from 10 weeks to 16 years, and given that the CA assessment age ranged from age 11 to age 22, no conclusions are warranted regarding timing effects or critical developmental windows.

Whereas all studies that performed mediation analyses were considered to have a sufficiently large sample size, one of the 13 studies that conducted moderation analyses may have had an insufficient sample size. This moderation study failed to find significant moderation effects for the two tested RFs [emotional and behavioral reward reactivity (41)]. In sum, the majority of the reviewed studies seemed to be appropriate in terms of statistical power. However, shortcomings raising the possibility of type I errors are that: (a) not all studies were underpinned by resilience-focused hypotheses (11), (b) some RFs were secondary findings, (c) most RFs were only significant in one study, and (d) some positive findings were not replicated with different combinations of CA and psychopathology.

Regarding the studied designs, we only included cohort designs in which the RF was assessed before psychopathology and CA was measured no later than the RF. This design criterion was of major importance, as it ensured a causal timeline according to which psychopathology at the time of the outcome assessment would less likely have affected the RF and the RF would less likely have affected the CA experience. However, a more advanced design would have been to also assess the RFs prior to the occurrence of CA, so that baseline levels of the RFs could have been taken into account. This would have allowed us to draw more stringent conclusions regarding which RFs are specific to mental health resilience after CA, and which RFs are time-independent and are predictive for mental health resilience regardless of being measured prior to or after CA. Similarly, if psychopathology would also have been measured prior to or together with CA, conclusions could have been drawn regarding the development of mental ill-health following CA, taking into account the baseline psychopathological level. Notably, some of the reviewed studies did control for baseline psychopathology levels. In sum, future research should investigate which of the RFs that predict mental health resilience are specific to the time period after the CA experience and which RFs are time-independent. Moreover, future research should not only examine the effectiveness of RFs in reducing the risk of psychopathology following CA, but should also examine the effectiveness of RFs in reducing the risk of the development of psychopathology following CA.

Critics might further raise the concern that our review does not capture resilience dynamics, given that most of the reported studies assessed the RFs at a single point in time. Yet, we believe that although the effectiveness of RFs may fluctuate, the RFs alter the relationship between CA and psychopathology irrespective of the time of their assessment, as long as they are measured after the occurrence of CA and prior to the assessment of psychopathology.

Overall, the review should be viewed in the light of the heterogeneity of the included studies (i.e., follow-up length, sample size, CA assessment age range, CA/ RF/ psychopathology assessment method, number of CA/ RF/ psychopathology types assessed per study, applied analysis techniques). Therefore, we do not claim that the supported RFs are protective following every type of CA, for every type of psychopathology, for individuals of all cultures, or at all developmental stages. In other words, it may potentially be the case that some of the reviewed RFs are supportive in one, but not in another context. For example, low levels of expressive suppression (i.e., low levels of suppressing emotions) may be protective in safe environments, but may be ineffective or perhaps even disadvantageous in highly dangerous and hazardous environments. As we reviewed 42 different RFs following 15 different forms of CA in an attempt to predict at least one out of nine different types of psychopathology, we ask the readers to be aware that our results are based on averages and may not generalize to all contexts, especially not when those are extreme and/ or exceptional. Yet, we conjecture that the supported RFs might be potential targets for alleviating the relationship between CA and psychopathology in young people. Nonetheless, replication research is critically needed to investigate the generalizability of RFs between people and across situations.

The fact that only two reviewed RFs were significant in more than one study, additionally highlights the crucial need of replication studies. In sum, future research should (a) replicate RF findings, (b) further examine community-level RFs, (c) study RF fluctuations as well as critical windows, and (d) scrutinize the therapeutic effectiveness of RF enhancement. Moreover, we advocate for more research along the lines of systemic resilience theories, to integrate individual-, family- and community-level RFs into one overall model. Along these lines, we believe that our review indicates that RFs do not function in isolation, but are connected via complex interrelations that eventually mediate and/ or moderate the relationship between CA and psychopathology.

In sum, this is the first preregistered systematic review on social, cognitive, emotional and behavioral RFs that attenuate psychopathology in young people after CA. The review revealed evidence for 20 amenable RFs. Interventions that improve the levels of these RFs may reduce the probability of psychopathology following CA. Clinicians could therefore look to improve these RFs as part of their focused intervention plans. The review provided support for a systemic framework of mental health resilience, as the identified RFs functioned on individual-, family- and community- levels. Moreover, our findings underpinned the notion that RFs function as complex interrelated systems. Therefore, we encourage resilience researchers to scrutinize RFs based on a systemic framework and to explore RFs as a complex interrelated system.

## Author contributions

PW, A-LvH, and JF were responsible for the study conception and the development of the study protocol. The literature screening (*N* = 1969) was conducted by AdG, HC, and JF. The literature re-screening (*N* = 220) was conducted by AdG and JF. Data extraction and quality ratings were conducted by AdG and JF. In case of disagreement or uncertainty PW was included in the discussion. *Post hoc* quality ratings for statistical analyses were conducted by PW and JF. JF led the conduction process of the review under the supervision of PW and A-LvH. The writing up was performed by JF under the supervision of PW and A-LvH. All authors contributed to and approved the final manuscript. PW was responsible for the funding of the review.

### Conflict of interest statement

The authors declare that the research was conducted in the absence of any commercial or financial relationships that could be construed as a potential conflict of interest.

## Abbreviations

CA: Childhood Adversity
RF(s): Resilience Factor(s)
DSM: Diagnostic and Statistical Manual of Mental Disorders
SES: Socio-Economic Status
MRA(s): Multiple Regression Analysis(es)
SEM(s): Structural Equation Model(s).
