# Supplementary material for: A Systematic Review of Amenable Resilience Factors That Moderate and/or Mediate the Relationship Between Childhood Adversity and Mental Health in Young People
Source: Front Psychiatry. 2018 Jun 19;9:230. doi: 10.3389/fpsyt.2018.00230 (PMC6018532; doi:10.3389/fpsyt.2018.00230)
Supplement: Supplementary file 1 [file Data_Sheet_1.pdf]

Supplementary Material

A Systematic Review of Amenable Resilience Factors that Moderate and/or Mediate the Relationship between Childhood Adversity and Mental Health in Young People

Jessica Fritz*, Anne M. de Graaff, Helen Caisley, A.-L. van Harmelen[A] & Paul O. Wilkinson[A]

*** Correspondence:** Jessica Fritz: jf585@cam.ac.uk

[A] **Shared last authorship**

1 Supplementary Description

1.A

The inter-rater reliability (IRR) calculation was based on nominal agreement ratings (1 = correct, 0 = incorrect), and was conducted in R with the 'epiR' package (1). Given that the data indicated prevalence problems (i.e. marginal distribution of the agreement ratings were substantially more often "correct" than "incorrect" ,2), we utilized Byrt's prevalence-adjusted kappa (3). We calculated Byrt's kappa separately for every abstracted article and averaged the revealed kappas.

1.B

We report the gender ratio for the baseline assessment or the investigated sample. If such information was not available we report information for the first reported assessment time. We report the average, not the absolute, study length and in cases of uncertainty we report the minimal length of the studies. We report the sample sizes which have been used for the analyses. We report the mean ages for the CA assessments, dependent on availability either for the full or for the investigated sample. CA assessment mean ages were provided for 12 of the 22 studies.

1.C

Notably, ego over-control mediated the association between early child maltreatment and alcohol use negatively (4). A lower level of early child maltreatment was associated with less ego over-control and a low level of ego over-control was in turn associated with more alcohol use (4). Given the negative mediation, we did not consider this effect as RF.

1.D

Notably, adolescent-father communication negatively mediated the association between paternal alcohol abuse problems and violation of rules, in girls (5). A lower level of CA was associated with more adolescent-father communication and more adolescent-father communication was in turn associated with more violation of rules (5). Given the negative mediation effect, we did not consider this effect as RF.

2 Supplementary Table

The data extraction form was partially underpinned by the STROBE report recommendations (6). The data extraction form covers the following topics: (a) Type of cohort design, (b) length of follow-up period (including time intervals), (c) definition of CA (theoretical and statistical scale used), (d) sample size (if possible per group and assessment occasion), (e) gender, (f) age, (g) demographics (i.e. socio-economic status & ethnicity), (h) measurement type (e.g. questionnaire/interview/objective measure), assessment instrument, and assessment time point for CA, (i) measurement type, assessment instrument, and assessment time point for RF(s), (j) measurements type, assessment instrument, and assessment time point for psychopathology (PP), (k) type (i.e. design/statistics/other) and definition for controlled confounders, (l) used statistics and statistical outcome (i.e. moderation/mediation, analysis method, analysis coefficient, analysis statistic, significance value, confidence interval/standard error, effect size), and (m) conclusion (significance and if appropriate directionality of the effect).

Data extraction item content template.

Item	Item content
**1**	Reference
**2**	ID
**3**	Note
**4**	Design
**5**	Note Design
**6**	Amount of assessment waves
**7**	Either indicate the time point for each assessment wave (e.g. T1 January 2010, T2 March 2013; or: T1 age 14, T2 age 17) or the incubation time between the assessments (e.g. T1: baseline, T2: T1 + 3 years, T3: T1 + 5 years)
**8**	Gender
**9**	CA definition
**10**	CA measurement
**11**	If CA dichotomous: define control group
**12**	Sample size (if possible per occasion; e.g. T1 = 600, T2 = 490)
**13**	If CA dichotomous: Sample size per group (and if possible per occasion; e.g. CA: T1 = 320, T2 = 170; no-CA: T1 = 500, T2 = 400)
**14**	Mean age (if possible per occasion; e.g. T1 = 14.5, T2 = 17.1)
**15**	Note age
**16**	If CA dichotomous: Mean age per group (and if possible per occasion; e.g. CA: T1 = 14.2, T2 = 17.0; no-CA: T1 = 14.8, T2 = 17.6)
**17**	Demographics: indicate time point (e.g. baseline)
**18**	Gender ratio
**19**	If CA dichotomous: Gender ratio per group (e.g. CA: 64% female; no-CA: 53% female)
**20**	SES
**21**	If CA dichotomous: SES per group (e.g. CA: low; no-CA: moderate)
**22**	Ethnicity
**23**	If CA dichotomous: Main Ethnicity per group (e.g. CA: 80% white, 20% mixed; no-CA: 90% white, 10% mixed)
**24**	CA assessment method
**25**	Name of CA assessment instrument (e.g. Childhood Trauma Questionnaire)
**26**	CA assessed at wave(s):
**27**	Amount of RFs
**28**	RF 1 assessment method
**29**	Name of RF 1
**30**	Name of RF 1 assessment instrument (e.g. Rosenberg Self-Esteem Scale)
**31**	RF 1 assessed at wave(s):
**32**	RF 2 assessment method
**33**	Name of RF 2
**34**	Name of RF 2 assessment instrument (e.g. Rosenberg Self-Esteem Scale)
**35**	RF 2 assessed at wave(s):
**36**	RF 3 assessment method
**37**	Name of RF 3
**38**	Name of RF 3 assessment instrument (e.g. Rosenberg Self-Esteem Scale)
**39**	RF 3 assessed at wave(s):
**40**	Amount of PP measures
**41**	PP assessment method 1
**42**	Name of PP 1
**43**	Name of PP assessment instrument 1 (e.g. Beck’s Depression Inventory)
**44**	PP 1 assessed at wave(s):
**45**	PP assessment method 2
**46**	Name of PP 2
**47**	Name of PP assessment instrument 2 (e.g. Beck’s Depression Inventory)

Supplement II to be continued.

Item	Item content
**48**	PP 2 assessed at wave(s):
**49**	PP assessment method 3
**50**	Name of PP 3
**51**	Name of PP assessment instrument 3 (e.g. Beck's Depression Inventory)
**52**	PP 3 assessed at wave(s):
**53**	Confounders measured and controlled for by: Design, statistics, or other (specify in description)
**54**	Specify confounders (e.g. SES, age, gender, etc.)
**55**	Specify PP:
**56**	Specify RF:
**57**	Choose: Mediation or Moderation
**58**	Analysis method e.g. regression, SEM, GEE, multilevel model + (MODERATION: (if neccesarry amount of RF main effects), amount of moderator(s), amount of RF moderator(s); MEDIATORS: amount of mediator(s), amount of RF mediator(s))
**59**	Analysis coefficient (e.g. beta)
**60**	Analysis statistic (e.g. t or z)
**61**	Significance (e.g. p value)
**62**	CI or SE
**63**	Effect Size
**64**	Note
**65**	Specify PP:
**66**	Specify RF:
**67**	Choose: Mediation or Moderation
**68**	Analysis method e.g. regression, SEM, GEE, multilevel model + (MODERATION: (if neccesarry amount of RF main effects), amount of moderator(s), amount of RF moderator(s); MEDIATORS: amount of mediator(s), amount of RF mediator(s))
**69**	Analysis coefficient (e.g. beta)
**70**	Analysis statistic (e.g. t or z)
**71**	Significance (e.g. p value)
**72**	CI or SE
**73**	Effect Size
**74**	Note
**75**	Specify PP:
**76**	Specify RF:
**77**	Choose: Mediation or Moderation
**78**	Analysis method e.g. regression, SEM, GEE, multilevel model + (MODERATION: (if neccesarry amount of RF main effects), amount of moderator(s), amount of RF moderator(s); MEDIATORS: amount of mediator(s), amount of RF mediator(s))
**79**	Analysis coefficient (e.g. beta)
**80**	Analysis statistic (e.g. t or z)
**81**	Significance (e.g. p value)
**82**	CI or SE
**83**	Effect Size
**84**	Note
**85**	Specify PP:
**86**	Specify RF:
**87**	Choose: Mediation or Moderation
**88**	Analysis method e.g. regression, SEM, GEE, multilevel model + (MODERATION: (if neccesarry amount of RF main effects), amount of moderator(s), amount of RF moderator(s); MEDIATORS: amount of mediator(s), amount of RF mediator(s))
**89**	Analysis coefficient (e.g. beta)
**90**	Analysis statistic (e.g. t or z)
**91**	Significance (e.g. p value)
**92**	CI or SE
**93**	Effect Size
**94**	Note
**95**	Specify PP:
**96**	Specify RF:
**97**	Choose: Mediation or Moderation
**98**	Analysis method e.g. regression, SEM, GEE, multilevel model + (MODERATION: (if neccesarry amount of RF main effects), amount of moderator(s), amount of RF moderator(s); MEDIATORS: amount of mediator(s), amount of RF mediator(s))
**99**	Analysis coefficient (e.g. beta)
**100**	Analysis statistic (e.g. t or z)
**101**	Significance (e.g. p value)
**102**	CI or SE
**103**	Effect Size
**104**	Note
**105**	Specify PP:
**106**	Specify RF:
**107**	Choose: Mediation or Moderation
**108**	Analysis method e.g. regression, SEM, GEE, multilevel model + (MODERATION: (if neccesarry amount of RF main effects), amount of moderator(s), amount of RF moderator(s); MEDIATORS: amount of mediator(s), amount of RF mediator(s))
**109**	Analysis coefficient (e.g. beta)
**110**	Analysis statistic (e.g. t or z)
**111**	Significance (e.g. p value)
**112**	CI or SE

Supplement II to be continued.

Item	Item content
**113**	Effect Size
**114**	Note
**115**	Analysis 1: Conclusion
**116**	Analysis 2: Conclusion
**117**	Analysis 3: Conclusion
**118**	Analysis 4: Conclusion
**119**	Analysis 5: Conclusion
**120**	Analysis 6: Conclusion
**121**	Name of RF 1
**122**	To which resilience factor category belongs the RF 1?
**123**	To which resilience factor domains belongs RF 1? NB: Indicate **ALL** domains that are applicable: e = emotional, b = behavioural, s = social, c = cognitive. E.g.: e, s.
**124**	Name of RF 2
**125**	To which resilience factor category belongs the RF 2?
**126**	To which resilience factor domains belongs RF 2? NB: Indicate **ALL** domains that are applicable: e = emotional, b = behavioural, s = social, c = cognitive. E.g.: e, s.
**127**	Name of RF 3
**128**	To which resilience factor category belongs the RF 3?
**129**	To which resilience factor domains belongs RF 3? NB: Indicate **ALL** domains that are applicable: e = emotional, b = behavioural, s = social, c = cognitive. E.g.: e, s.

3 Supplementary Table

Downs and Black's (7) scale is recommended for the assessment of randomized, as well as non-randomized studies (8). Given that we exclusively evaluated cohort studies we excluded 9 of 27 items, which are specific to randomized studies (i.e. adverse intervention effects, quality of the description of the used intervention, same recruitment for all intervention groups, representativeness of treatment facilities, intervention compliance, intervention blinding, randomization procedure, randomization concealment and power ,7). We duplicated one item, which assesses the accuracy of the outcome measure (i.e. psychopathology), twice, to also assess the accuracy of the CA and RF variables. Accordingly, the adapted quality rating scale contained 20 items.

Quality assessment item content template: Adapted version of Downs and Black's (7) *quality rating scale.*

Item	Item content<br>***NB: content of the original items from Downs and Black's*** (7) ***scale can be retrieved from (i.e. see appendix): http://jech.bmj.com/content/jech/52/6/377.full.pdf***
**Article**	
**1**	Reference
**2**	ID
**3**	Note
**Description (1 = yes, 0 = no)**<br>***NB: Questions which start with a * have another rating system***	
**4**	original item 1
**5**	original item 2
**6**	original item 3
**7**	original item 5* 2 = yes, 1 = partially, 0 = no
**8**	original item 6
**9**	original item 7
**10**	original item 9
**11**	original item 10
**Validity (1 = yes, 0 = no, 000 = unable to determine)**	
**12**	original item 11
**13**	original item 12
**14**	original item 15 (In our case: This is about whether those measuring psychopathology outcome were blind to the resilience factor(s).)
**15**	original item 16
**16**	original item 17 (In our case: Are the analyses adjusted for differences between participants regarding the length of follow-up?)
**17**	original item 18
**18**	adaption of the original item 20 (In our case: Was the childhood adversity measure used valid and reliable?)
**19**	adaption of the original item 20 (In our case: Were the resilience factor measures used valid and reliable?)
**20**	original item 20 (In our case: Were the psychopathology measures used valid and reliable?)
**21**	original item 21 (In our case: Were the participants in different CA groups recruited from the same population?)
**22**	original item 25
**23**	original item 26
**Total score**	
**24**	NB: This is an automatic field please do not enter numbers manually!

4 Supplementary Table

Analysis Method Quality Assessment: Item Content Template

Item	Item content
**Article**	
**1**	Reference
**2**	ID
**Sample Size**	
**3**	Sample size
**4**	Appropriateness sample size: Moderation (NA = no moderation performed , 0 = inappropriate, 1 = appropriate)
**5**	Appropriateness sample size: Mediation (NA = no mediation performed , 0 = inappropriate, 1 = appropriate)
**RFs tested**	
**6**	How many RFs tested in total?
**7**	How many RFs significant in total?
**8**	How many RFs significant moderators?
**9**	How many RFs significant mediators?
**10**	Name of significant RFs
**Single Versus Multiple RFs**	
**11**	Multiple or single RF models?
**12**	Amount of moderators per model (0 if not appropriate; i.e. only RF moderators are counted)
**13**	Amount of mediators per model (0 if not appropriate; i.e. only RF mediators are counted)
**Quality Moderation Analysis**	
**14**	Moderation Rating (NA = no moderation performed, ? = not rateable, 1 = no/visual inspection, 2 = correlational post hoc probing, 3 = regression post hoc probing)
**15**	Note: Moderation
**Quality Mediation Analysis**	
**16**	Mediation Rating (NA = no mediation performed, ? = not rateable, 1 = no/direct effect reduction to non-significance, 2 = Sobel (or comparable formulas), 3 = bootstrap)
**17**	Note: Mediation
**Multiple Testing**	
**18**	Correction for multiple testing? (Not Neccesary = NN, no = 0, yes =1)
**19**	Note: Correction for multiple testing?

5 Supplementary Description

In order to obtain a sample size guideline for interaction effects we conducted a power analysis. The resilience literature shows that whereas multifaceted clusters of CAs explain up to 30 percent of psychopathology indices (9), RF predictors have small to at the best moderate effect sizes (10). Moreover, research has indicated that interaction effects in social sciences are generally weak (11–13). For example, Champoux and Peters (12) reviewed 23 studies and found that moderation effects account for approximately 1 to 3 percent of the outcome variable (mean ΔR^2 = .03). Similarly, Aguinis and colleagues (11) found that the 261 reviewed moderation analyses had an average interaction effect size of .01 (f^2). Based on those findings and on the fact that many of the reviewed studies include additional covariates or interaction effects, which requires larger sample sizes, we calculated the sample size for a moderation analysis with a moderate total effect (f^2 = .15). The analysis was conducted in R with the package ‘pwr’ (14). We specified two main effects, one interaction effect (u = 3), a moderate effect size of f^2 = .15 (i.e. based on the above described findings and on Cohen’s effect size criteria ,15), an alpha level of .05, and a power threshold of .80. The analysis showed that a minimum of 77 participants is required (v = 72.71; *N* = v + (u+1); *N* = 72.71 + 4) to detect a moderate effect.

6 Supplementary Table

Studied Types of Childhood Adversity and Psychopathology.

CHILDHOOD ADVERSITY			
**Childhood Maltreatment**	**Intra-Family Adversity**	**Community Adversity**	**Clustered Life Adversities**
emotional abuse(4,16–20)	marital distress/conflict(21)	ethnic-political conflict(22–24)	adverse life experiences(25,26)
sexual abuse(4,17,18,27,28)	parental problem drinking(5)	community violence(29)	
physical abuse(4,17,18,20,27,30)	aggressive parenting behaviour(31)		
emotional neglect(4,20)	parental violence(32)		
physical neglect(4,20)	parental mental health problems(33)		
	stressful family-level life events(34)		
	accumulated family adversity(35)		

PSYCHOPATHOLOGY	
**Disorder Types**	**Clustered Types of Psychopathology**
anxiety symptoms(16,17,24)/ social anxiety symptoms(19)	psychological distress(25,28)
depressive symptoms(17,19,24,27,31,33,35)	mental well-being(21,28)
posttraumatic stress symptoms(18,22,23)	externalizing(4,5,21,23,29,30)
substance (ab)use symptoms(4,32,34)	internalizing(4,21,23,29,30)
conduct symptoms(20,24,26)	

REFERENCES

1. Stevenson M. Package "epiR": Tools for the Analysis of Epidemiological Data. R package version 0.9-79. (2016) Available at: https://cran.r-project.org/web/packages/epiR/index.html
2. Hallgren KA. Computing Inter-Rater Reliability for Observational Data: An Overview and Tutorial. *Tutor Quant Methods Psychol* (2012) **8**:23–34.
3. Byrt T, Bishop J, Carlin JB. Bias, Prevalence and Kappa. *J Clin Epidemiol* (1993) **46**:423–429.
4. Oshri A, Rogosch FA, Cicchetti D. Child Maltreatment and Mediating Influences of Childhood Personality Types on the Development of Adolescent Psychopathology. *J Clin Child Adolesc Psychol* (2013) **42**:287–301. doi:10.1080/15374416.2012.715366
5. Finan LJ, Schulz J, Gordon MS, McCauley Ohannessian C. Parental problem drinking and adolescent externalizing behaviors: The mediating role of family functioning. *J Adolesc* (2015) **43**:100–110. doi:10.1016/j.adolescence.2015.05.001
6. Vandenbroucke JP, von Elm E, Altman DG, Gøtzsche PC, Mulrow CD, Pocock SJ, Poole C, Schlesselman JJ, Egger M, STROBE Initiative. Strengthening the Reporting of Observational Studies in Epidemiology (STROBE): Explanation and Elaboration. *Epidemiology* (2007) **18**:805–835. doi:10.1097/EDE.0b013e3181577511
7. Downs SH, Black N. The feasibility of creating a checklist for the assessment of the methodological quality both of randomised and non-randomised studies of health care interventions. *J Epidemiol Community Health* (1998) **52**:377–384. doi:10.1136/jech.52.6.377
8. Deeks JJ, Dinnes J, D'Amico R, Sowden AJ, Sakarovitch C, Song F, Petticrew M, Altman DG, International Stroke Trial Collaborative Group, European Carotid Surgery Trial Collaborative Group. Evaluating non-randomised intervention studies. *Health Technol Assess (Rockv)* (2003) **7**:1–173.
9. Kessler RC, McLaughlin KA, Greif Green J, Gruber MJ, Sampson NA, Zaslavsky AM, Aguilar-Gaxiola S, Alhamzawi AO, Alonso J, Angermeyer M, et al. Childhood adversities and adult psychopathology in the WHO World Mental Health Surveys. *Br J Psychiatry* (2010) **197**:378–385. doi:10.1192/bjp.bp.110.080499
10. Kalisch R, Baker DG, Basten U, Boks MP, Bonanno GA, Brummelman E, Chmitorz A, Fernàndez G, Fiebach CJ, Galatzer-Levy I, et al. The resilience framework as a strategy to combat stress-related disorders. *Nat Hum Behav* (2017) **1:**784–90. doi:10.1038/s41562-017-0200-8
11. Aguinis H, Beaty JC, Boik RJ, Pierce CA. Effect Size and Power in Assessing Moderating Effects of Categorical Variables Using Multiple Regression: A 30-Year Review. *J Appl Psychol* (2005) **90**:94–107. doi:10.1037/0021-9010.90.1.94
12. Champoux JE, Peters WS. Form, effect size and power in moderated regression analysis. *J Occup Psychol* (1987) **60**:243–255. doi:10.1111/j.2044-8325.1987.tb00257.x
13. Fairchild AJ, McQuillin SD. Evaluating mediation and moderation effects in school psychology: A presentation of methods and review of current practice. *J Sch Psychol* (2010) **48**:53–84. doi:10.1016/j.jsp.2009.09.001.
14. Champely S. pwr: Basic Functions for Power Analysis. R package version 1.2-1. (2017) Available at: https://cran.r-project.org/package=pwr
15. Cohen J. *Statistical power analysis for the behavioral sciences.* 2nd ed. Hillsdale, NJ: Lawrence Erlbaum Associates (1988).
16. Banducci AN, Lejuez CW, Dougherty LR, MacPherson L. A Prospective Examination

of the Relations Between Emotional Abuse and Anxiety: Moderation by Distress Tolerance. *Prev Sci* (2017) **18**:20–30. doi:10.1007/s11121-016-0691-y
17. Hankin BL. Childhood Maltreatment and Psychopathology: Prospective Tests of Attachment, Cognitive Vulnerability, and Stress as Mediating Processes. *Cognit Ther Res* (2005) **29**:645–671. doi:10.1007/s10608-005-9631-z
18. Walter KH, Horsey KJ, Palmieri PA, Hobfoll SE. The Role of Protective Self-cognitions in the Relationship Between Childhood Trauma and Later Resource Loss. *J Trauma Stress* (2010) **23**:264–273. doi:10.1002/jts.20504
19. Calvete E. Emotional abuse as a predictor of early maladaptive schemas in adolescents: Contributions to the development of depressive and social anxiety symptoms. *Child Abuse Negl* (2014) **38**:735–746. doi:10.1016/j.chiabu.2013.10.014
20. You S, Lim SA. Development pathways from abusive parenting to delinquency: The mediating role of depression and aggression. *Child Abuse Negl* (2015) **46**:152–162. doi:10.1016/j.chiabu.2015.05.009
21. Cui M, Conger RD. Parenting Behavior as Mediator and Moderator of the Association Between Marital Problems and Adolescent Maladjustment. *J Res Adolesc* (2008) **18**:261–284. doi:10.1111/j.1532-7795.2008.00560.x
22. Dubow EF, Huesmann LR, Boxer P, Landau S, Dvir S, Shikaki K, Ginges J. Exposure to Political Conflict and Violence and Posttraumatic Stress in Middle East Youth: Protective Factors. *J Clin Child Adolesc Psychol* (2012) **41**:402–416. doi:10.1080/15374416.2012.684274
23. Qouta S, El-Sarraj E, Punamäki R-L. Mental flexibility as resiliency factor among children exposed to political violence. *Int J Psychol* (2001) **36**:1–7. doi:10.1080/00207590042000010
24. Shahar G, Henrich CC. Perceived Family Social Support Buffers Against the Effects of Exposure to Rocket Attacks on Adolescent Depression, Aggression, and Severe Violence. *J Fam Psychol* (2015) **30**:163–168. doi:10.1037/fam0000179
25. Boyes ME, Hasking PA, Martin G. Adverse Life Experience and Psychological Distress in Adolescence: Moderating and Mediating Effects of Emotion Regulation and Rumination. *Stress Heal* (2016) **32**:402–410. doi:10.1002/smi.2635
26. Masten AS, Hubbard JJ, Gest SD, Tellegen A, Garmezy N, Ramirez M. Competence in the context of adversity: Pathways to resilience and maladaptation from childhood to late adolescence. *Dev Psychopathol* (1999) **11**:143–169.
27. Dennison MJ, Sheridan MA, Busso DS, Jenness JL, Peverill M, Rosen ML, McLaughlin KA. Neurobehavioral Markers of Resilience to Depression Amongst Adolescents Exposed to Child Abuse. *J Abnorm Psychol* (2016) **125**:1201–1212. doi:10.1037/abn0000215
28. Hébert M, Cénat JM, Blais M, Lavoie F, Guerrier M. Child Sexual Abuse, Bullying, Cyberbullying, and Mental Health Problems Among High Schools Students: A Moderated Mediated Model. *Depress Anxiety* (2016) **33**:623–629. doi:10.1002/da.22504
29. Hardaway CR, Sterrett-Hong E, Larkby CA, Cornelius MD. Family Resources as Protective Factors for Low-Income Youth Exposed to Community Violence. *J Youth Adolesc* (2016) **45**:1309–1322. doi:10.1007/s10964-015-0410-1
30. Lansford JE, Malone PS, Stevens KI, Dodge KA, Bates JE, Pettit GS. Developmental trajectories of externalizing and internalizing behaviors: Factors underlying resilience in physically abused children. *Dev Psychopathol* (2006) **18**:35–55. doi:10.1017/S0954579406060032
31. Gaté MA, Watkins ER, Simmons JG, Byrne ML, Schwartz OS, Whittle S, Sheeber LB, Allen NB. Maternal Parenting Behaviors and Adolescent Depression: The

Mediating Role of Rumination. *J Clin Child Adolesc Psychol* (2013) **42**:348–357. doi:10.1080/15374416.2012.755927
32. Jester JM, Steinberg DB, Heitzeg MM, Zucker RA. Coping Expectancies, Not Enhancement Expectancies, Mediate Trauma Experience Effects on Problem Alcohol Use: A Prospective Study From Early Childhood to Adolescence. *J Stud Alcohol Drugs* (2015) **76**:781–789. doi:10.15288/jsad.2015.76.781
33. Klasen F, Otto C, Kriston L, Patalay P, Schlack R, Ravens-Sieberer U, The BELLA study group. Risk and protective factors for the development of depressive symptoms in children and adolescents: results of the longitudinal BELLA study. *Eur Child Adolesc Psychiatry* (2015) **24**:695–703. doi:10.1007/s00787-014-0637-5
34. Hicks BM, Johnson W, Durbin CE, Blonigen DM, Iacono WG, McGue M. Delineating Selection and Mediation Effects Among Childhood Personality and Environmental Risk Factors in the Development of Adolescent Substance Abuse. *J Abnorm Child Psychol* (2014) **42**:845–859. doi:10.1007/s10802-013-9831-z
35. van Harmelen A-L, Gibson JL, St Clair MC, Owens M, Brodbeck J, Dunn V, Lewis G, Croudace T, Jones PB, Kievit RA, et al. Friendships and Family Support Reduce Subsequent Depressive Symptoms in At-Risk Adolescents. *PLoS One* (2016) **11**:e0153715. doi:10.1371/journal.pone.0153715
